# 

**DOI:** 10.1192/bjb.2019.22

**Published:** 2019-08

**Authors:** Catherine Marshall, Ania Korszun

**Affiliations:** Clinical Lecturer in Psychiatry, Centre for Psychiatry, Queen Mary University of London, UK; Professor of Psychiatry and Education, Barts and The London School of Medicine and Dentistry, Queen Mary University of London, UK. Email: a.korszun@qmul.ac.uk

This is a comprehensive resource for professionals working with students in higher education. Subsections of the book address in turn the student experience, caring for students with mental health issues and how to foster mental health in distinct student populations. The book takes an academically robust approach to supporting student self-care, wellbeing and development of resilience.

Individual chapters go on to address key presentations around substance misuse, suicidality, mood and anxiety disorders, psychotic illness, autism, ADHD, trauma, sleep and eating disorders. There is also an important focus upon the role of university mental health services for students who have faced sexual violence, those from military backgrounds and first generation university attenders, as well as students identifying as part of the LGBTQ community. It is in the last chapter where the challenges for ‘medical students, residents and fellows’ are explored, albeit somewhat briefly. The writers identify the unique stressors for this group and reflect upon the obstacles to seeking help such as stigma and confidentiality. It is noteworthy that all medical students in the American system are postgraduate and therefore usually older than British students, most of whom enter medical school straight from sixth form/college and are less prepared for the expectations of professionalism at this early stage of their development.

Although written from the perspective of the American educational system, in general the content is still eminently transferrable to UK institutions. Throughout the book, its contributors make few assumptions as to prior knowledge, detailing everything from the risk-taking behaviours that develop during ‘emerging adulthood’ to the descriptive psychopathology for different major mental illnesses. Each chapter's utilisation of ‘key concept’ bullet points and case examples further increase its accessibility to the reader.

Ultimately the book makes recommendations not only on how student health programmes can achieve excellence by successfully managing students with major mental illness, but also on how all students can be supported to reach their full potential. It is a valuable resource for teachers in higher education.

